# Supplementary material for: Spatially-local inhibition and synaptic plasticity together enable dynamic, context-dependent integration of parallel sensory pathways
Source: bioRxiv. 2025 Oct 19:2025.10.18.683230. Preprint. [Version 1] doi: 10.1101/2025.10.18.683230 (PMC12632782; doi:10.1101/2025.10.18.683230)
Supplement: 1 [file NIHPP2025.10.18.683230V1-supplement-1.pdf]

bioRxiv preprint doi: https://doi.org/10.1101/2025.10.18.683230; this version posted October 19, 2025. The copyright holder for this preprint (which was not certified by peer review) is the author/funder, who has granted bioRxiv a license to display the preprint in perpetuity. It is made available under aCC-BY 4.0 International license.

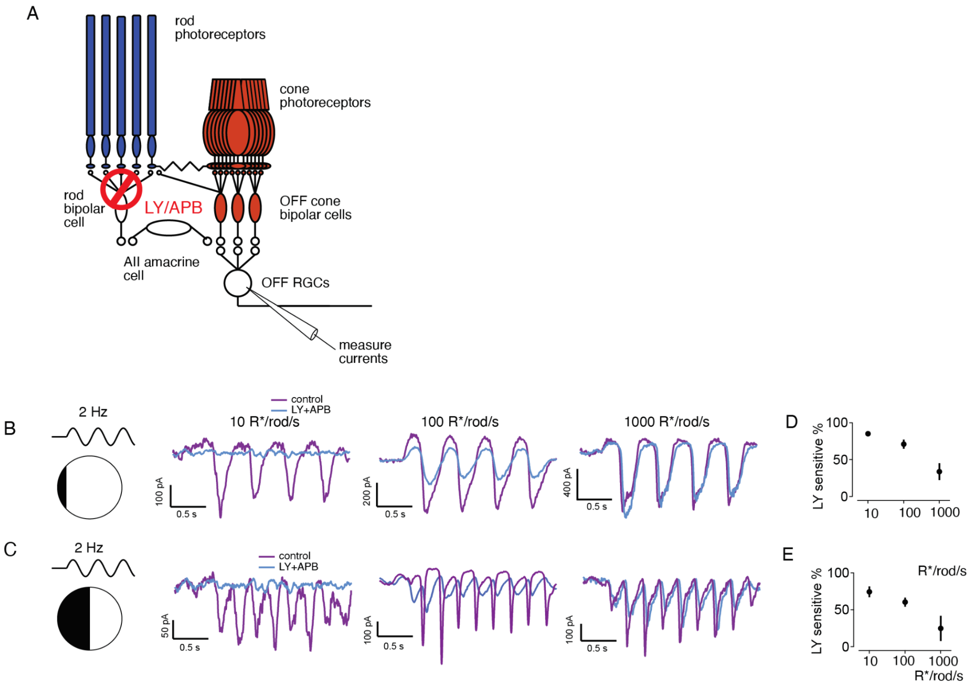

Supplementary Figure 1. Routing of Rod-derived signals under different light levels.

(A) Schematic of the experimental setup showing pharmacological isolation of rod pathways using LY/APB to block rod bipolar cell signaling in the primary rod pathway.

(B) Example current traces (control, purple; LY+APB treatment, blue) from OffT αRGC in response to a 2 Hz full-field modulating spots at 10 R*/rod/s, 100 R*/rod/s, and 1000 R*/rod/s.

(C) Similar current traces showing OffT αRGC responses to a contrast-reversing grating stimulus.

(D-E) Quantification of the LY/APB-sensitive component of the OffT αRGC responses at different light intensities (R*/rod/s).

bioRxiv preprint doi: https://doi.org/10.1101/2025.10.18.683230; this version posted October 19, 2025. The copyright holder for this preprint (which was not certified by peer review) is the author/funder, who has granted bioRxiv a license to display the preprint in perpetuity. It is made available under aCC-BY 4.0 International license.

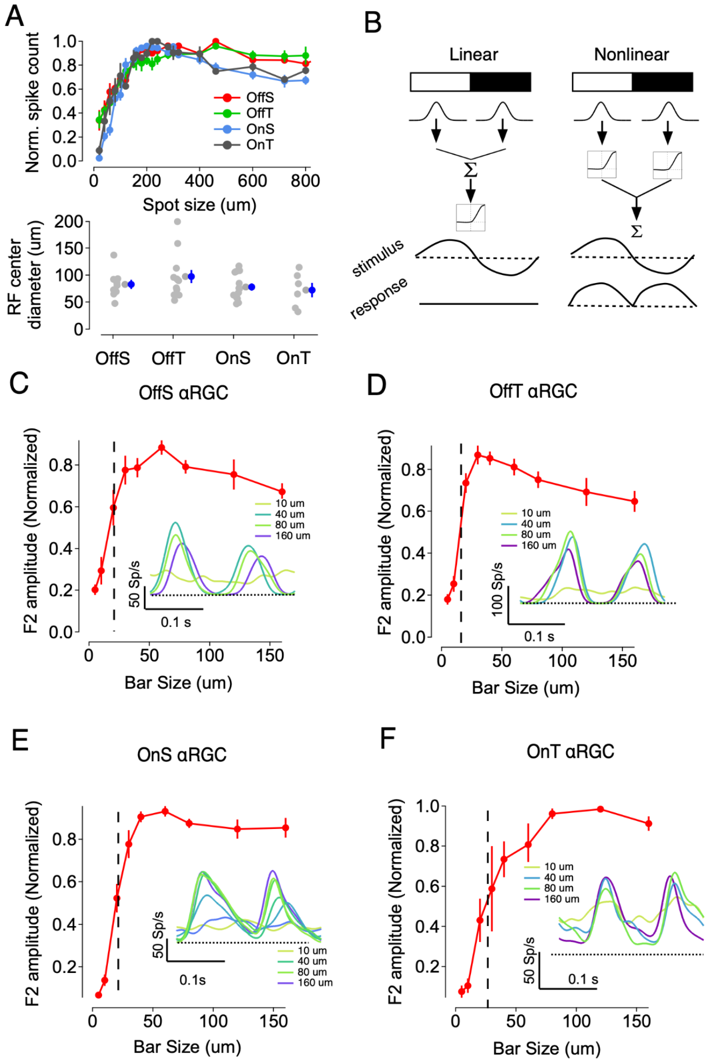

Supplementary Figure 2. Nonlinear subunits in αRGCs receptive field.

(A) Top: Area summation curves for four alpha retinal ganglion cell (αRGC) subtypes: Off-sustained (OffS), Off-transient (OffT), On-sustained (OnS), and On-transient (OnT). The summation curves plot the normalized response amplitude as a function of stimulus diameter

Bottom: Scatter plots show the distribution of RF sizes. Error bars represent mean ± SEM.

(B) Schematic illustration of the contrast-reversing grating stimulus used to probe the presence of nonlinear subunits in αRGC receptive fields. Nonlinear subunit receptive fields produce frequency-doubled (F2) responses when presented with such gratings, while linearly integrating receptive fields do not

(C-F) Population summary data showing the nonlinear F2 response amplitude as a function of bar width. Inset: cycle average responses in an example αRGC of respective subtype.

bioRxiv preprint doi: https://doi.org/10.1101/2025.10.18.683230; this version posted October 19, 2025. The copyright holder for this preprint (which was not certified by peer review) is the author/funder, who has granted bioRxiv a license to display the preprint in perpetuity. It is made available under aCC-BY 4.0 International license.

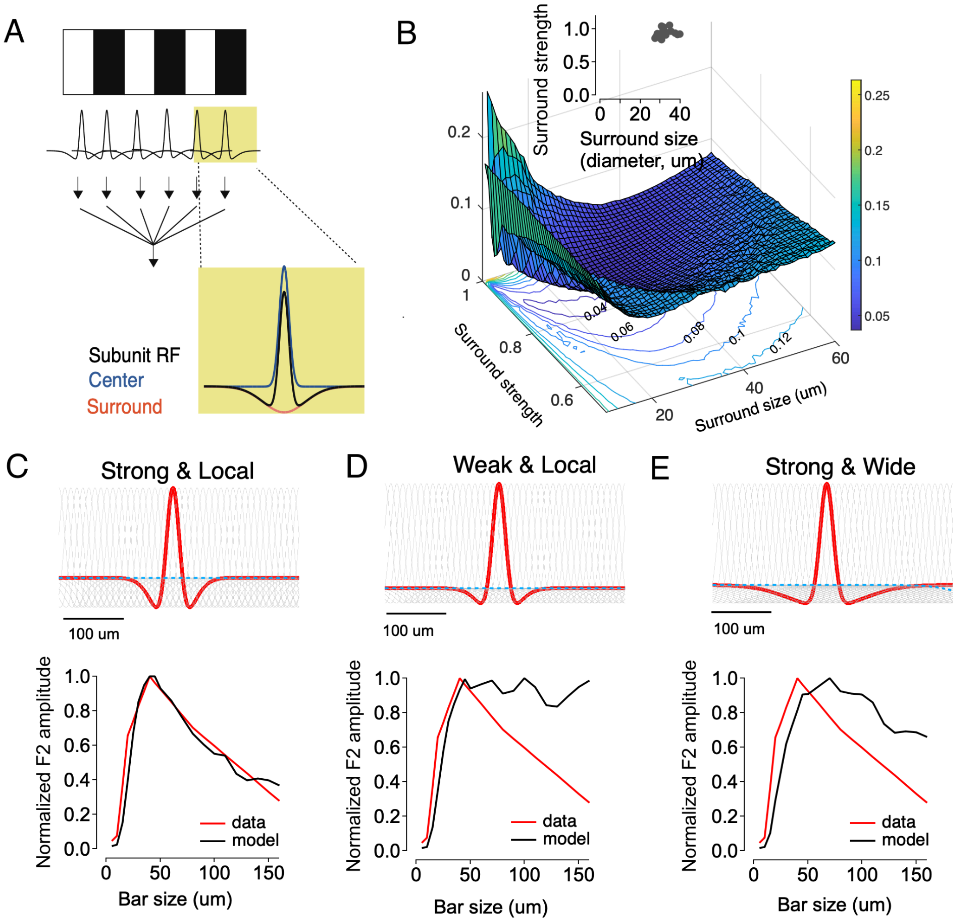

Supplementary Figure 3. Strong and local surround mediates spatial tuning of inhibitory subunits.

(A) Schematic of the center-surround subunit model for inhibitory inputs.

(B) Parameter space showing the effects of surround size and strength on model fit quality.

(C-E) Model fits (black) to experimental data (red) for inhibitory inputs with different surround properties: strong & local (C, surround size 15 μm, surround strength 0.9), weak & local (D surround size 15 μm, surround strength 0.2), and strong & nonlocal (E, surround size 40 μm, surround strength 0.9).

bioRxiv preprint doi: https://doi.org/10.1101/2025.10.18.683230; this version posted October 19, 2025. The copyright holder for this preprint (which was not certified by peer review) is the author/funder, who has granted bioRxiv a license to display the preprint in perpetuity. It is made available under aCC-BY 4.0 International license.

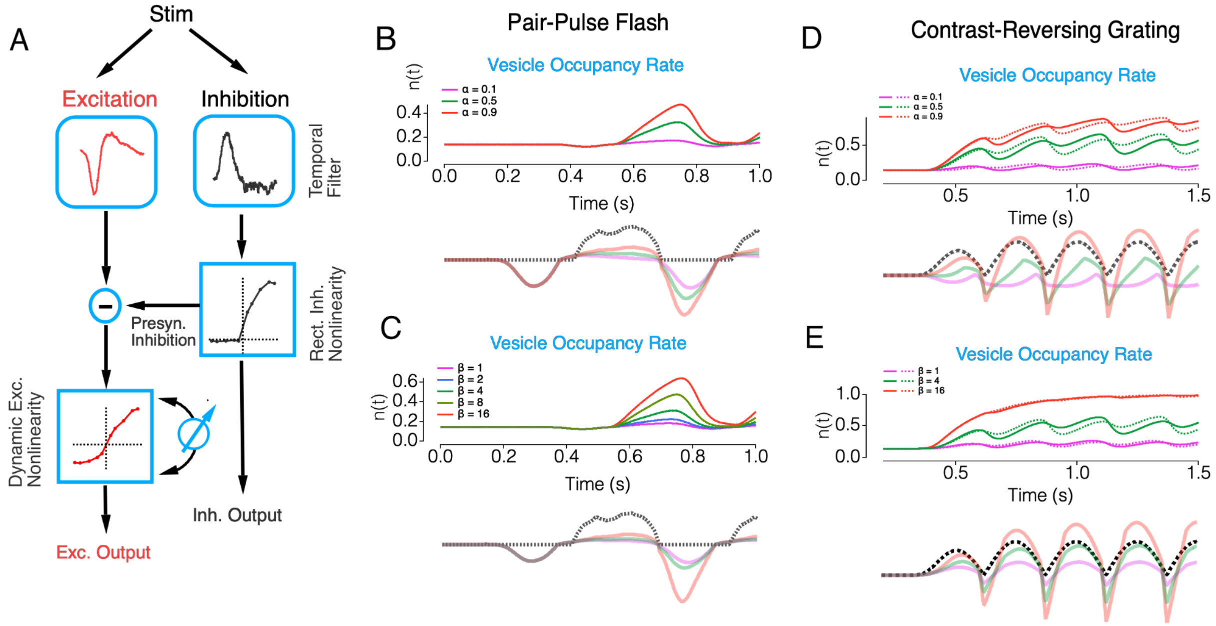

Supplementary Figure 4. Dynamic Synaptic Depression Mediates History-Dependent Excitation/Inhibition Interaction in Retinal Processing

(A) Schematic of the temporal dynamic synapse model showing the excitatory pathway (red) and inhibitory pathway (black). Inhibition modulates excitation through presynaptic inhibition, with inhibitory pathways passing through rectifying nonlinearity while excitatory pathways passing through piecewise nonlinearity. The blue arrow indicates the dynamic regulation of excitatory output through vesicle occupancy.

bioRxiv preprint doi: https://doi.org/10.1101/2025.10.18.683230; this version posted October 19, 2025. The copyright holder for this preprint (which was not certified by peer review) is the author/funder, who has granted bioRxiv a license to display the preprint in perpetuity. It is made available under aCC-BY 4.0 International license.

(B-C) Simulated responses to paired-pulse flash stimuli. Top panels show vesicle occupancy rate (n(t)) and bottom panels show excitatory conductance output (colored lines) along with inhibitory current (dashed black line). (B) demonstrates the effect of varying presynaptic inhibition strength (α) while (C) shows responses with different inhibition sensitivity values (β).
(D-E) Model responses to contrast-reversing grating stimuli.

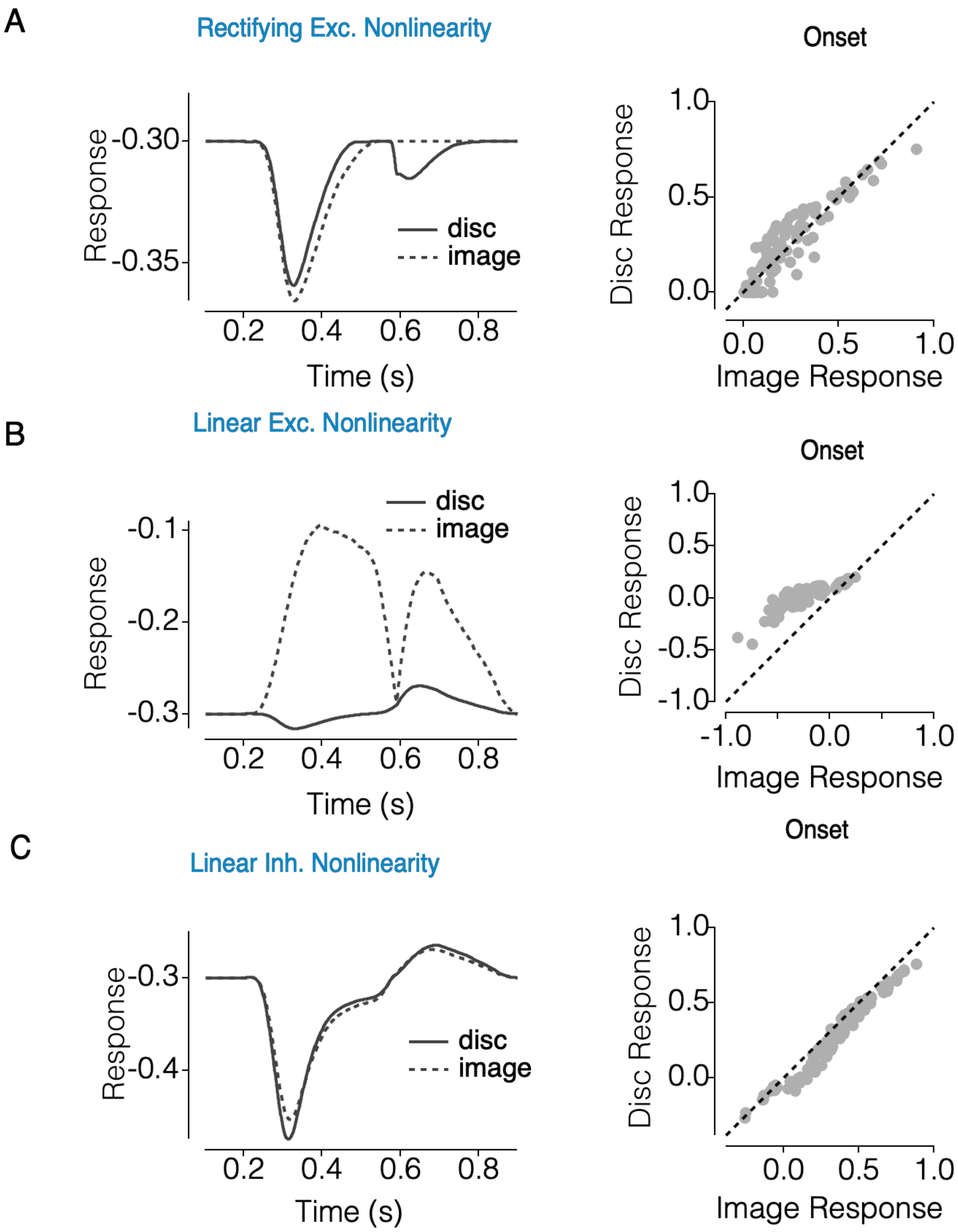

Supplementary Figure 5: Effects of excitatory and inhibitory nonlinearities on spatial integration in the retina

(A) Example response of the model with rectifying excitatory nonlinearity to natural image patches (dashed line) and their corresponding linear equivalent discs (solid line). Left panel shows example response traces over time. Right panel shows the scatter plot of image responses versus disc responses

bioRxiv preprint doi: https://doi.org/10.1101/2025.10.18.683230; this version posted October 19, 2025. The copyright holder for this preprint (which was not certified by peer review) is the author/funder, who has granted bioRxiv a license to display the preprint in perpetuity. It is made available under aCC-BY 4.0 International license.

at stimulus onset, with most points clustered along the unity line (dashed), indicating similar responses to both stimulus types.

(B) Response of the model with linear excitatory nonlinearity to the same stimuli. Left panel shows example traces demonstrating suppression of responses to natural images (dashed line) compared to disc stimuli (solid line). Right panel shows the scatter plot of responses at stimulus onset.

(C) Response of the model with linear inhibitory nonlinearity. Left panel shows example traces with similar responses to both natural images (dashed line) and linear equivalent discs (solid line). Right panel shows the scatter plot of responses at stimulus onset, with points clustered along the unity line, indicating that linear inhibition does not generate differential responses to spatial structure.

bioRxiv preprint doi: https://doi.org/10.1101/2025.10.18.683230; this version posted October 19, 2025. The copyright holder for this preprint (which was not certified by peer review) is the author/funder, who has granted bioRxiv a license to display the preprint in perpetuity. It is made available under aCC-BY 4.0 International license.

Supplementary Table 1. Parameters for the Temporal and Spatial-Temporal Dynamic Synapse Models

Parameter	Description	Temporal Only Model Value	Spatial-Temporal Model Value	Unit
K_{rec}	Vesicle recovery rate	10	10	Hz
K_{rel}	Vesicle release rate	5	5	Hz
b	Release gain	5	5	-
γ	Rectification ratio (exc)	0.3	0.3	-
E_0	Norm. excitation baseline	0.3	0.3	-
α	Presynaptic inhibition Strength [0,1]	0.9	0.9	-
β	Sensitivity of release rate to inhibition	1	1	-
E_0	Baseline excitation	0.3	0.3	-
σ_{exc}	Excitatory subunit center size	-	22	μm

bioRxiv preprint doi: https://doi.org/10.1101/2025.10.18.683230; this version posted October 19, 2025. The copyright holder for this preprint (which was not certified by peer review) is the author/funder, who has granted bioRxiv a license to display the preprint in perpetuity. It is made available under aCC-BY 4.0 International license.

Parameter	Description	Temporal Only Model Value	Spatial-Temporal Model Value	Unit
d_{exc}	Excitatory subunit spacing	-	44	μm
$\sigma_{pool,exc}$	Excitatory pooling radius	-	50	μm
σ_{inh}	Inhibitory subunit center size	-	12	μm
d_{inh}	Inhibitory subunit spacing	-	24	μm
$\sigma_{inh, surround}$	Inhibitory surround size	-	20	μm
δ	Surround strength onto inhibition subunit	-	0.9	-
$\sigma_{pool,inh}$	Inhibitory pooling radius	-	100	μm
